# Essential Oil Profiles of *Lippia turbinata* (Verbenaceae) from Argentina: Insights from a Systematic Review and Meta‐Analysis

**DOI:** 10.1002/open.202500203

**Published:** 2025-06-24

**Authors:** Fernando Livio Corzo, Franco Emmanuel Calvo, Emilio Fernando Lizarraga, Guillermo Emilio Marcial, Maria Ines Mercado

**Affiliations:** ^1^ Instituto de Ambiente de Montaña y Regiones Áridas Universidad Nacional de Chilecito Ruta Los Peregrinos s/n, Los Sarmientos Chilecito, La Rioja F5360CKB Argentina; ^2^ Consejo Nacional de Investigaciones Científicas y Técnicas (CONICET) Buenos Aires C1414 Argentina; ^3^ Facultad de Ciencias Naturales Universidad Nacional de Tucumán Miguel Lillo 205 Tucumán T4000JFD Argentina; ^4^ Fundación Miguel Lillo Miguel Lillo 251 Tucumán T4000JFD Argentina; ^5^ Instituto de Ciencia y Tecnología de Alimentos Córdoba (ICYTAC) CONICET‐UNC Dr. Juan Filloy s/n Córdoba X5000 Argentina

**Keywords:** chemical compositions, essential oils, intraspecific variabilities, multivariate analyses, poleo

## Abstract

*Lippia turbinata* (Verbenaceae), “poleo,” is an aromatic shrub native to Argentina, Chile, and Paraguay. Traditionally used as food and medicine, it exhibits a great polymorphism and essential oil (EO) variability. This study analyzes the EO profile of the different populations of *L. turbinata* distributed throughout Argentina through a meta‐analysis comparing their chemical composition of EOs reported in the literature. The meta‐analysis comparison with available literature suggests the existence of chemotypes within the species that may correlate with infraspecific genetic differences, geographical origin of the plants, phenological stage, soil, and stress conditions, among other factors. This analysis reveals that the most frequent and abundant compounds are limonene, carvone, β‐caryophyllene, and its derivative oxide. This approach of multivariate statistical analyses enables to compare EO profiles of different populations and identify critical thresholds for selecting chemotypes to optimize the species’ applications in the food, pharmacology, and agriculture industries.

## Graphical abstract

This study systematically reviews essential oil (EO) profiles of *Lippia turbinata* (Verbenaceae) populations from Argentina. A meta‐analysis reveals significant chemical variability and distinct chemotypes, primarily defined by limonene, carvone, β‐caryophyllene, and caryophyllene oxide. Insights may guide optimized uses of these EOs in food, pharmacology, and agroindustry.

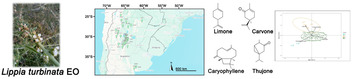

## 1 Introduction

The products of the secondary metabolism of plants are considered nonvital but necessary for survival. Their role is mainly related to the interaction with the environment as a defense against biotic or abiotic factors, attraction to pollinators, dispersers, or other beneficial organisms, and competition established in intra‐ or interspecific interactions.^[^ ^1^, ^2^ ^]^ Metabolomics is a discipline that arouses particular interest in the development of analytical methodologies for the determination of all chemical compounds synthesized in living organisms, which is called the metabolome. A subgroup of the metabolome is the volatilome, which includes the set of compounds that, due to their physicochemical properties, are volatile at room pressure and temperature. The determination of the volatile components of the plants is relevant for the study of plant physiology, for the survey of potential sources of aromas, and as a bioprospecting tool.^[^ ^3^ ^]^

Essential oils (EOs) are a major component of the volatilome, consisting of complex mixtures of volatile, lipophilic, and aromatic compounds commonly present in aromatic plants. EOs can contain between 20 and 60 components present in variable concentrations.^[^ ^4^ ^]^ These metabolites play an important role in biological interactions, attracting pollinators, and acting as antibacterial, antioxidant, antiviral, antifungal, insecticidal, and/or antifeedant agents.^[^ ^5^, ^6^ ^]^ The biosynthesis and chemical profile of EOs can be under genetic and/or environmental control, exhibiting variations in a certain species depending on the specimen location, infraspecific genetic differences, phenological stage of the plant, the type of soil, the conditions of water stress, and the season of the year, among other factors,^[^ ^6^, ^7^ ^]^ determining the existence of chemotypes.^[^ ^8^ ^]^ Additionally, the isolation method (such as advanced supercritical fluid extraction, subcritical extraction liquid, solvent‐free microwave extraction, or conventional hydrodistillation, steam distillation, hydrodiffusion, and solvent extraction) can also determine the composition, biological, and physicochemical properties of the isolated EO.^[^ ^9^ ^]^

Among aromatic plants, the Verbenaceae family stands out, featuring species primarily native to tropical and temperate regions of the Americas, with only a few representatives originating from the Old World. In Argentina, this family comprises ≈23 genera, among which *Aloysia*, *Glandularia*, *Verbena*, *Lantana*, *Lippia*, and *Acantholippia* are distinguished for their use in ornamental, food, and medicinal applications.^[^ ^10^, ^11^ ^]^

The *Lippia* genus has around 120 species, primarily distributed across the temperate, tropical, and subtropical regions of America, from Mexico to central Argentina, where about 26 species are found.^[^ ^10^ ^]^ Many *Lippia* species are valued for medicinal, food, cosmetic, and ornamental uses.^[^ ^12^, ^13^ ^]^

*Lippia turbinata* Griseb, popularly known as “poleo,” “té del país,” “té criollo,” or “poleo fino,” is native to mountainous regions of northwest and central Argentine (Jujuy, Salta, Tucumán, Catamarca, Santiago del Estero, La Rioja, Chaco, Córdoba, Buenos Aires, Mendoza, San Luis, San Juan, and La Pampa, in the phytogeographic provinces of Yungas, Monte, Espinal, and Chaco) and in the neighboring countries of Chile and Paraguay.^[^ ^10^ ^]^ Its aerial parts have a long tradition of use for food purposes (in the compound herb industry) and medicinal purposes (codified in the Argentine Pharmacopoeia—Res 2673/99) for the preparation of digestive, diuretic, tonic, and emmenagogue infusions and decoctions.^[^ ^11^, ^13^, ^14^ ^]^

A characteristic of the genus *Lippia*, and *L. turbinata*, is its great polymorphism associated with a high phytochemical variability at intraspecific level.^[^ ^15^ ^]^ Currently, two botanical forms of poleo are recognized, *L. turbinata* f. *turbinata* and *L. turbinata* f. *magnifolia* Moldenke (Syn. *L. fissicalyx* Tronc). Both are highly branched aromatic shrubs that can reach 1–2 m in height. In general, the forms differ morphologically by the length of their internodes, the presence or absence of brachyblasts, and the size and arrangement of the leaves. However, there are individuals with intermediate characteristics that make their identification difficult.^[^ ^10^ ^]^

Although the mono‐ and sesquiterpenoids identified in the EO of *Lippia* species are common and widespread across the plant kingdom, a distinctive characteristic of this genus is the significant variation in the EO composition reported for the same species from different geographic origins.^[^ ^16^ ^]^ There are numerous studies on the EO of *L. turbinata*,^[^ ^17^, ^18^, ^19^, ^20^, ^21^, ^22^, ^23^, ^24^, ^25^, ^26^, ^27^, ^28^, ^29^, ^30^, ^31^, ^32^, ^33^, ^34^ ^]^ revealing qualitative and quantitative differences as well as variations in yield.^[^ ^15^, ^16^ ^]^

The aim of this work was to compile and compare the chemical composition of the EO profiles of *L. turbinata* populations distributed throughout Argentina, which are reported in scientific literature. This work seeks to identify patterns of chemical variability across different geographic regions by performing a comprehensive meta‐analysis of the compiled data. Special attention was given to the identification of distinct chemotypes and populations exhibiting unique or particularly noteworthy profiles of volatile compounds. Through this analysis, the study aims to contribute to a deeper understanding of the species’ phytochemical diversity and its potential applications in pharmacology, aromatherapy, and agriculture.

## 2 Data Collection and Statistical Analysis

**Figure 1:**
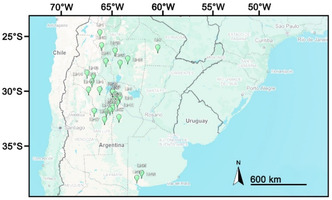
Geographical distribution of the 32 *L. turbinata* populations selected from published literature sources. Copyright 2025, Google Earth.

Data on the chemical characteristics of *L. turbinata* EO were collected from the literature using academic search engines for scientific articles and academic resources, such as Google Scholar, PubMed, or Scopus. The search was conducted using different combinations of keywords, including “essential oil,” *“Lippia turbinata*,” and “Argentina,” covering the period from 1990 to the present. This yielded over 400 related publications. After a thorough evaluation, many studies were excluded due to being review articles, lacking scientific rigor, or missing essential information such as the isolation process, geographic location, or chemical profile. Ultimately, 32 *L. turbinata* populations selected for analysis are summarized in **Table** **1** and **Figure** **1**. The EO data, including isolation method, gas chromatography–mass spectrometry (GC–MS) analytical characteristics, quantity and type of metabolites present, yield, location, and date (phenological state) of the sample, were tabulated and analyzed using algorithms for statistical description. Multivariate data analysis using principal component analysis (PCA) and Ward's hierarchical clustering method based on compositional distance matrices and hierarchical cluster analysis to indicate affinity groups was performed using the software R Project for Statistical Computing software.^[^ ^35^ ^]^

**Table 1:** Summary of available literature on *Lippia turbinata* essential oil from different collection sites.

*ID*	Reference	Site	Major compounds (≥5%)	EO yield (%)	Extraction method, equipment, and conditions of analysis
Lt‐1	Leal et al., 2018^[^ ^25^ ^]^	Salta	Limonene (84.3%) ß‐Caryophyllene (6.1%)	No data	Hydrodistillation 4 h and Perkin Elmer Series Clarus 600 gas chromatograph equipped with a flame ionization detector (FID), Clarus 600 T mass spectrometer detector, and a DB‐5 MS fused‐silica capillary column (60 m × 0.25 mm × 0.25 μm).
Lt‐2	García et al., 2018 (1)^[^ ^32^ ^]^	Characato I Córdoba	Limonene (60.8%) Bornyl acetate (8.2%) Carvone (5.8%)	No data	Hydrodistillation and Perkin Elmer Clarus 600 GC–MS instrument equipped with two columns: Supelcowax 10 (30 m× 0.25 μm) and DB‐5 fused‐silica column (30 m × 0.25 μm).
Lt‐3	García et al., 2018 (2)^[^ ^33^ ^]^	Characto II Córdoba	Limonene (62.1%) Bornyl acetate (8.4%) Carvone (6.0 %)	No data	Hydrodistillation and Perkin Elmer Clarus 600 GC–MS (Waltham, Massachusetts, United States) instrument equipped with two columns: Supelcowax 10 (30 m × 0.25 mm) and DB‐5 fused‐silica column (30 m × 0.25 mm).
Lt‐4	Gleiser and Zygadlo, 2007^[^ ^23^ ^]^	Olta La Rioja	(α)‐Thujone (48.3%) Carvone (17.4%) ß‐Caryophyllene (10.0%)	No data	Hydrodistillation (2 h) and gas–liquid chromatography and MS on a Perkin Elmer Q‐700 equipment. A polar column of 30 m × 0.25 mm × 0.25 μm (CBwax) and an apolar column of 30 m × 0.25 mm × 0.25 μm (DB‐5).
Lt‐5	Quiroga et al., 2013^[^ ^27^ ^]^	Altas Cumbres Córdoba	Limonene (76.8%) 1,8‐Cineole (4.8%)	No data	Hydrodistillation (2 h) and GC–MS in a Perkin Elmer Q‐700 equipment coupled with an ion trap mass detector (Perkin Elmer, Shelton, CT, USA). A column DB‐5 (30 m × 0.25 mm i.d. and 0.25 μm coating thickness).
Lt‐6	Barbieri et al., 2016^[^ ^24^ ^]^	Antinaco Valley‐Los Colorados La Rioja	Piperitenone oxide (63.0%) Limonene (7.2%) Caryophyllene oxide (5.6%)	1.2	Hydrodistillation (3 h) and GC–MS carried out on a Shimadzu GC‐2010 GC–MS‐QP2010 (Shimadzu Corporation, Kyoto, Japan) fitted with a DB 5‐fused‐silica column (30 m × 0.25 mm i.d. and film thickness 0.25 μm).
Lt‐7	Perez Zamora et al., 2016^[^ ^28^ ^]^	Universidad Nacional de Chaco Chaco	Carvone (80.7%) Limonene (8.7%)	No data	Hydrodistillation and by gas chromatography and mass spectrometry (GC/MS) in a Clarus 600 equipment, Perkin Elmer (Instituto Multidisciplinario de Biología Vegetal Universidad Nacional de Córdoba), under the following conditions: DB5 column (60 m × 0.25 mm i.d. × 0.25 μm).
Lt‐8	Pellegrini et al., 2017^[^ ^29^ ^]^	Los Molinos Córdoba	*trans*‐Sabinol (58.3%) (α)‐Thujone (14.2%) Sabinene (8.2%)	No data	Hydrodistillation and Shimadzu 2010 GC coupled to a Shimadzu QP2010 plus mass spectrometer (MS).
Lt‐9	Velasco Negueruela et al., 1993^[^ ^17^ ^]^	Colón Córdoba	(α)‐Thujone (28.3%) Caryophyllene oxide (8.0%) Carvone (7.4%)	1	Hydrodistillation and a Hewlett‐Packard 5890 gas chromatograph fitted with a fused‐silica SE‐54 capillary column (50 m × 0.125 mm). The chromatograph was coupled to an HP 5971 A mass selective detector at 70 eV.
Lt‐10	Zygadlo et al., 1995^[^ ^18^ ^]^	Saldan Córdoba	(α)‐Thujone (30.2%) Carvone (10.1%) Limonene (6.1%)	0.1	Hydrodistillation and Hewlett‐Packard 5890 gas chromatograph fitted with a 30 m × 0.25 mm fused‐silica SE‐54 capillary column. The chromatograph was coupled to a HP 5971 A mass selective detector at 70 eV.
Lt‐11	Dellacasa et al., 2003^[^ ^19^ ^]^	San Luis	Limonene (43.3%) Piperitenone oxide (24.8%) 1,8 Cineole (14.7%)	1.3	Hydrodistillation and no data.
Lt‐12	Duschatzky et al., 2004^[^ ^20^ ^]^	Merlo, San Luis	Limonene (60.6%) Piperitenone oxide (17.8%) ß‐Caryophyllene (6.4%)	1.3	Hydrodistillation (4 h) and GC/MS analyses were carried out on a GC‐HP 6890 with a mass selective detector (quadrupole) HP 5973, ionization energy 70 eV, fitted with an HP‐5MS column (5% phenyl methyl‐siloxane, 30 m × 0.25 mm, and film thickness 0.25 μm).
Lt‐13	Juliani et al., 2004^[^ ^21^ ^]^	Los Llanos La Rioja	Limonene (48.1%) Piperitenone oxide (30.1%) Spathulenol (6.7%)	1.1	Steam distillation and GC coupled to an MS and FID (Agilent GC System 6890 Series, Mass Selective Detector, Agilent 5973 Network, FID detector).
Lt‐14	Corzo et al., 2024^[^ ^34^ ^]^^]^	Chilecito La Rioja	Limonene (48.9%) Piperitenone oxide (22.9%) 1,8 Cineole (7.11%)	1.2	Hydrodistillation (2 h) and GC/MS analyses by Hewlett‐Packard 6890 gas chromatograph coupled to a Hewlett‐Packard 5973 quadrupole mass spectrometer equipped with a Perkin Elmer Elite‐5MS capillary column (5% phenylmethylsiloxane, 30 m × 0.25 mm i.d., and film thickness 0.10 μm) and a mass selective detector were used for MS detection. Ionization energy at 70 eV. Helium was taken as carrier gas with a flow rate of 1.0 mL min. The temperature of the injector and ion source were set at 230 and 280 ºC, respectively. The injection volume was 1 μL with a split ratio of 80:1 and the oven temperature was programmed as follows: held at 60 ºC for 1 min, then increasing temperature from 60 to 185 °C at 1.5 °C min, held at 185 °C during 1 min, rising again from 185 to 275 ºC at 9 ºC min and finally held at 275 ºC during 2 min.
Lt‐15	Retta et al., 2024^[^ ^30^ ^]^	San Agustín del Valle Fértil San Juan	Limonene (30.8%) Piperitenone oxide (29.9%) *cis*‐ Piperitenone epoxide (13.2%)	1.8	Hydrodistillation and CG‐FID‐MS using a Perkin Elmer Clarus 500 equipment with a special configuration: an automatic injector, a splitter of two columns of different polarity, the polar one coupled to a FID detector and the nonpolar one divided into 2 flows by means of an MS‐Vent system toward a FID detector and a quadrupole detector.
Lt‐16	Retta et al., 2024^[^ ^30^ ^]^	Suncho Corral Stgo del Estero	Limonene (46.0%) Piperitenone oxide (17.3%) 1,8‐Cineole (8.1%)	0.9	Hydrodistillation and CG‐FID‐MS using a Perkin Elmer Clarus 500 equipment with a special configuration: an automatic injector, a splitter of two columns of different polarity, the polar one coupled to a FID detector and the nonpolar one divided into 2 flows by means of an MS‐Vent system toward a FID detector and a quadrupole detector.
Lt‐17	Retta et al., 2024^[^ ^30^ ^]^	Sierra de las quijadas San Luis	Piperitenone oxide (48.5%) Limonene (32.2%)	2.4	Hydrodistillation and CG‐FID‐MS using a Perkin Elmer Clarus 500 equipment with a special configuration: an automatic injector, a splitter of two columns of different polarity, the polar one coupled to a FID detector and the nonpolar one divided into 2 flows by means of an MS‐Vent system toward a FID detector and a quadrupole detector.
Lt‐18	Retta et al., 2024^[^ ^30^ ^]^	Los Lobos San Luis	Limonene (39.4%) Piperitenone oxide (25.1 %) Piperitenone (7.3%)	1.2	Hydrodistillation and CG‐FID‐MS using a Perkin Elmer Clarus 500 equipment with a special configuration: an automatic injector, a splitter of two columns of different polarity, the polar one coupled to a FID detector and the nonpolar one divided into 2 flows by means of an MS‐Vent system toward a FID detector and a quadrupole detector.
Lt‐19	Retta et al., 2024^[^ ^30^ ^]^	Dique paso de las carretas San Luis	Limonene (18.0%) Caryophyllene oxide (11.4%) 1,8‐Cineole (10.2%)	0.3	Hydrodistillation and CG‐FID‐MS using a Perkin Elmer Clarus 500 equipment with a special configuration: an automatic injector, a splitter of two columns of different polarity, the polar one coupled to a FID detector and the nonpolar one divided into 2 flows by means of an MS‐Vent system toward a FID detector and a quadrupole detector.
Lt‐20	Retta et al., 2024^[^ ^30^ ^]^	Villa del Soto I Córdoba	Carvone (62.0%) Limonene (22.8%)	1.8	Hydrodistillation and CG‐FID‐MS using a Perkin Elmer Clarus 500 equipment with a special configuration: an automatic injector, a splitter of two columns of different polarity, the polar one coupled to a FID detector and the nonpolar one divided into 2 flows by means of an MS‐Vent system toward a FID detector and a quadrupole detector.
Lt‐21	Retta et al., 2024^[^ ^30^ ^]^	Villa del Soto II Córdoba	Piperitenone oxide (45.9%) Limonene (28.7%)	1.3	Hydrodistillation and CG‐FID‐MS using a Perkin Elmer Clarus 500 equipment with a special configuration: an automatic injector, a splitter of two columns of different polarity, the polar one coupled to a FID detector and the nonpolar one divided into 2 flows by means of an MS‐Vent system toward a FID detector and a quadrupole detector.
Lt‐22	Retta et al., 2024^[^ ^30^ ^]^	Travesia Córdoba (commercial sample purchased in market)	Piperitenone oxide (42.9%) Limonene (31.6%)	1.1	Hydrodistillation and CG‐FID‐MS using a Perkin Elmer Clarus 500 equipment with a special configuration: an automatic injector, a splitter of two columns of different polarity, the polar one coupled to a FID detector and the nonpolar one divided into 2 flows by means of an MS‐Vent system toward a FID detector and a quadrupole detector.
Lt‐23	Retta et al., 2024^[^ ^30^ ^]^	Rio Pinto Córdoba	Caryophyllene oxide (25.8%) Spathulenol (7.4%) *p*‐Cymene (7%)	0.2	Hydrodistillation and CG‐FID‐MS using a Perkin Elmer Clarus 500 equipment with a special configuration: an automatic injector, a splitter of two columns of different polarity, the polar one coupled to a FID detector and the nonpolar one divided into 2 flows by means of an MS‐Vent system toward a FID detector and a quadrupole detector.
Lt‐24	Retta et al., 2024^[^ ^30^ ^]^	Bahía Blanca Buenos Aires	Limonene (39.7%) Piperitenone (23.9%) Piperitenone oxide (7.1%)	1.4	Hydrodistillation and CG‐FID‐MS using a Perkin Elmer Clarus 500 equipment with a special configuration: an automatic injector, a splitter of two columns of different polarity, the polar one coupled to a FID detector and the nonpolar one divided into 2 flows by means of an MS‐Vent system toward a FID detector and a quadrupole detector.
Lt‐25	Retta et al., 2024^[^ ^30^ ^]^	Saldungaray II Buenos Aires	Limonene (30.5%) Piperitenone (20.1%) 1,8‐cineole (9.8%)	1.5	Hydrodistillation and CG‐FID‐MS using a Perkin Elmer Clarus 500 equipment with a special configuration: an automatic injector, a splitter of two columns of different polarity, the polar one coupled to a FID detector and the nonpolar one divided into 2 flows by means of an MS‐Vent system toward a FID detector and a quadrupole detector.
Lt‐26	Retta et al., 2024^[^ ^30^ ^]^	Saldungaray I Buenos Aires	Limonene (23.1%) Piperitenone (19.3%) Piperitenone oxide (10.9%)	1.5	Hydrodistillation and CG‐FID‐MS using a Perkin Elmer Clarus 500 equipment with a special configuration: an automatic injector, a splitter of two columns of different polarity, the polar one coupled to a FID detector and the nonpolar one divided into 2 flows by means of an MS‐Vent system toward a FID detector and a quadrupole detector.
Lt‐27	Passone and Etcheverry, 2014^[^ ^26^ ^]^	Río Cuarto Córdoba (commercial sample purchased in market)	Limonene (48.8%) β‐Caryophyllene epoxide (18.1%) Piperitenone (7.7%)	1.02	Steam distillation and a Perkin Elmer Clarus 600 equipped with a 60 m × 0.25 mm i.d. and film thickness 0.25 μm, DB5 Perkin Elmer column.
Lt‐28	Coronel et al., 2005^[^ ^22^ ^]^	El Naranjito Santiago del Estero	Limonene (69.0%) Piperitenone (7.2%)	1.3	Hydrodistillation and GC–MS analysis were carried out on a 5973 Hewlett‐Packard selective mass detector coupled to a Hewlett‐Packard 6890 GC using a capillary HP‐5MS (5% phenylmethylsiloxane).
Lt‐29	Coronel et al., 2005^[^ ^22^ ^]^	Dique Los Molinos Córdoba	Caryophyllene oxide (17.8%) Limonene (11.9%) Spathulenol (11.4%)	0.2	Hydrodistillation and GC–MS analysis were carried out on a 5973 Hewlett‐Packard selective mass detector coupled to a Hewlett‐Packard 6890 GC using a capillary HP‐5MS (5% phenylmethylsiloxane).
Lt‐30	Coronel et al., 2005^[^ ^22^ ^]^	Santa María Catamarca	Limonene (43.9%) Piperitenone oxide (40.4%)	0.2	Hydrodistillation and GC–MS analysis were carried out on a 5973 Hewlett‐Packard selective mass detector coupled to a Hewlett‐Packard 6890 GC using a capillary HP‐5MS (5% phenylmethylsiloxane).
Lt‐31	Coronel et al., 2005^[^ ^22^ ^]^	Ruta. Nac. 38, Km 62, Catamarca	Carvone (50.9%) Limonene (14.4%) Piperitenone oxide (14.0%)	no data	Hydrodistillation and GC–MS analysis were carried out on a 5973 Hewlett‐Packard selective mass detector coupled to a Hewlett‐Packard 6890 GC using a capillary HP‐5MS (5% phenylmethylsiloxane).
Lt‐32	Tapia Matar et al., 2024^[^ ^31^ ^]^	Loreto, Santiago del Estero	Limonene (66.7%) 1,8‐cineole (13.1%)	2.4	Hydrodistillation and GC‐FID analysis were carried out on a GC Konik 3000 series equipped with a ZB‐5 capillary column (30 m × 0.25 mm × 0.25 μm) (Phenomenex, Inc., Torrance, CA, USA) and a FID. And also a Thermo Scientific Focus GC coupled with a DSQ II electron ionization mass detector using an A TR‐5MS capillary column (30 m × 0.25 mm × 0.25 μm) (Thermo Fisher Scientific).

PCA was particularly crucial in reducing the dimensionality of the complex dataset, allowing for the visualization and interpretation of the major trends and patterns in EOs composition. By transforming the original correlated variables into a set of orthogonal components, PCA highlighted the primary sources of variation among the samples, making it easier to identify chemotypes and groupings related to geographical origin, developmental stage, or environmental factors. This statistical approach provided a robust framework to detect underlying relationships and differentiate between the chemical profiles of *L. turbinata*, facilitating a more informed assessment of its phytochemical diversity and potential applications.

## 3 *Lippia turbinata,* Essential Oil Variations, and its Main Constituents

**Figure 2:**
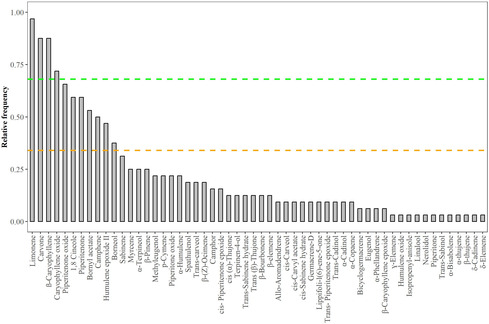
Relative frequency of occurrence of the different components reported for the EO of different populations of *L. turbinata* available in the literature. The green dashed line indicates the cutoff of compounds with high relative frequency of occurrence (above 0.68), while the yellow dashed line separates compounds with medium and low frequencies of occurrence.

**Figure 3:**
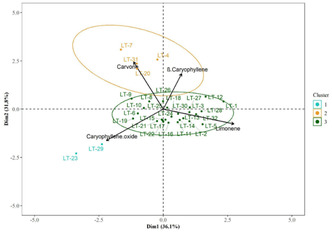
PCA score plot generated with the four components with major relative frequency of occurrence for the EO of different populations of *L. turbinata* available in the literature.

An analysis of the relative frequency of occurrence of the different analytes in the EO of *L. turbinata* populations reported in the literature revealed that the most frequent and abundant compounds are the monoterpene limonene, the oxygenated monoterpene carvone, the sesquiterpene β‐caryophyllene, and its derivative oxide. In contrast, compounds such as piperitenone, piperitenone oxide, 1,8‐cineole, bornyl acetate, camphene, humulene epoxide II, and borneol displayed intermediate levels of relative abundance or frequency of occurrence among the populations. The remaining 45 metabolites exhibited a low frequency of occurrence (**Figure** **2** and Supplementary Table 1). Additionally, depending on the population examined, α‐thujone, bornyl acetate, *trans*‐sabinol, or caryophyllene oxide have also occasionally been reported as major components (**Figure** **3**).

The PCA, using the four compounds with the highest relative frequency of occurrence (limonene, carvone, β‐caryophyllene, and caryophyllene oxide) as variables, explains 67.9% of the total variability exhibited in the EO profiles among the studied populations. The analysis groups the different populations into three clusters, showing no correlation with the collection site, phenological stage, or the extraction and analysis methods used. However, it supports clustering for groups I and II based on the presence and abundance of major compounds. *Cluster I* included Lt‐23 and 29, characterized by EO profiles dominated by caryophyllene oxide, the oxidized derivative of caryophyllene (Figure 3).

**Caryophyllene**, also known as β‐caryophyllene or *trans*‐caryophyllene, is a bicyclic sesquiterpene commonly found in plant EOs, known for its spicy and woody aroma as well as its bioactive properties. It is unique among terpenes due to its ability to interact with the endocannabinoid system, specifically as a CB2 receptor agonist, exhibiting excellent anti‐inflammatory, antioxidant, and immunomodulatory effects. These properties highlight its therapeutic potential, particularly for managing depression and anxiety.^[^ ^36^ ^]^ Additionally, caryophyllene exhibits antimicrobial, antioxidant, and gastroprotective activities, further enhancing its pharmacological potential.^[^ ^37^, ^38^ ^]^ Its derivative, caryophyllene oxide, offers enhanced oxidative stability, making it valuable in food, cosmetic, and pharmaceutical formulations. Preclinical studies have shown that caryophyllene oxide exhibits cytotoxic effects on certain cancer cell lines.^[^ ^39^ ^]^ Furthermore, it has exhibited insect‐repellent and insecticidal properties, positioning it as a sustainable option for pest management.^[^ ^38^ ^]^ Caryophyllene and its derivatives are considered safe for use under regulated usage conditions, emphasizing their potential in diverse applications.

*Cluster II* compromised populations Lt‐4, 7, 20, and 31, where **carvone** (unspecified enantiomer) was identified as either a primary or secondary major compound (Figure 3). Notably, Lt‐4 exhibited (α)‐thujone as the dominant compound, while carvone was the major constituent in Lt‐7, 20, and 31, with limonene as the second most abundant compound. Carvone naturally occurs as two enantiomers: dextrorotatory (d‐) and levorotatory (l‐), each eliciting different biological responses to olfactory receptors. S‐(+)‐carvone (or d‐carvone) has a mentholated, spicy aroma with rye‐like notes and medium strength, while R‐(−)‐carvone (or l‐carvone) is characterized by a minty and sweetish odor. Plants rich in carvone are extensively used in the cosmetic and food industries as preservatives and flavorings.^[^ ^40^ ^]^ In the pharmaceutical industry, it exhibits a range of biological activities, including antibacterial, antifungal, antiparasitic, antineuraminidase, antioxidant, anti‐inflammatory, and anticancer properties.^[^ ^41^, ^42^ ^]^ In agriculture, carvone is valued for its allelopathic properties and as antimicrobial, insecticidal, or repellent agent.^[^ ^43^ ^]^ It has also been evaluated as an antisprouting agent during tuber storage.^[^ ^40^ ^]^ At standard formulation concentrations, carvone is widely recognized as a safe natural preservative, bioactive compound, compound, and flavoring ingredient safe for use in food and cosmetics, with regulatory approvals from organizations such as the U. S. Food and Drug Administration (FDA) and the European Food Safety Authority. However, when used as a pesticide, further evaluations of carvone's degradation products in soil, the environment, and living organisms are necessary, as comprehensive risk assessments remain limited.^[^ ^44^, ^45^ ^]^

Since **limonene** is often one of the main components of the EO from *L. turbinata,* in addition to its characteristic aroma, it has a wide range of pharmacological properties and low toxicity that make it attractive to be incorporated into medical and pharmaceutical formulations.^[^ ^15^ ^]^ Limonene is one of the most abundant monoterpenes in nature and exists in two enantiomeric forms: D‐limonene [R‐(+)‐limonene], characterized by its citrus aroma, and L‐limonene [S‐(‐)‐limonene], which has a pine‐like scent.^[^ ^46^ ^]^ Due to its pleasant fragrance, low toxicity, and versatile bioactivity, D‐limonene has found widespread applications in the food, pharmaceutical, and cosmetic industries.^[^ ^47^ ^]^ It is approved as a food additive and flavoring agent in many countries and is listed as Generally Recognized As Safe by the FDA.^[^ ^46^ ^]^ However, its industrial and therapeutic applications require adherence to established safety protocols. A review of the pharmacology of limonene in *Lippia* species and *L. turbinata* reports the following properties: antimicrobial, antioxidant, anti‐inflammatory, antinociceptive, anticancer, antidiabetic, antihyperalgesic, antiviral, gastroprotective, and insecticidal.^[^ ^25^, ^27^ ^]^

*Cluster III* did not display a marked dominance of any of the four compounds of highest relative abundance; moreover, the group exhibited a high intragroup variability, including Lt‐1‐3, 5‐6, 8‐19, 21‐22, 24‐28, 30, and 32. Among these, Lt‐1‐3, 5, 11‐19, 21‐22, 24‐28, 30, and 32 had limonene as the major or second major compound, while piperitenone oxide was dominant in Lt‐6, 17, 21, and 22. Populations Lt‐9 and 10 exhibited (α)‐thujone as major metabolites. Lt‐8 uniquely lacked limonene, with *trans*‐sabinol and *s*(α)‐thujone as its primary constituents (Figure 3).

**Piperitenone**, one of the main components of several collections, is an α, β‐di‐unsaturated ketone and a precursor of piperitenone oxide (= lippione) and piperitone.^[^ ^48^ ^]^ It has been reported as an insecticidal, larvicidal, antiviral, and antitumoral agent.^[^ ^49^ ^]^ Its derivative, piperitenone oxide, exhibits a broad spectrum of bioactivities, including cardiovascular, bradycardic, hypotensive, antinociceptive, analgesic, insecticidal, trypanocidal, schistosomicidal, antimicrobial, and larvicidal activities.^[^ ^25^, ^50^ ^]^ Piperitenone oxide‐rich EOs may serve as natural insecticides, offering significant potential for use in eco‐friendly formulations.^[^ ^51^ ^]^ However, the regulatory assessment of EOs containing piperitenone oxide is ongoing.

Their use in food and cosmetic products requires further evaluation to establish safe concentration limits and minimize potential risks.^[^ ^39^ ^]^

**Thujones**, detected in populations Lt‐4 (cluster II), 9 and 10 (cluster III), and α‐thujone.^[^ ^17^, ^18^, ^23^, ^29^ ^]^ Thujones are bicyclic monoterpene ketones existing in two stereoisomeric forms: *cis‐ or* (α)‐thujone and *trans‐* or (β)‐thujone, where their proportions vary depending on the botanical source.^[^ ^52^ ^]^ Thujones are historically well‐known for their association with absinthe and are of interest in medicine, food science, and pharmacology due to their diverse biological activities. They exhibited antimicrobial, antifungal, and antioxidant properties, contributing to the preservative qualities of EOs. In traditional medicine, thujone‐containing plants have been used to stimulate digestion and appetite. Additionally, their insect‐repellent properties make them valuable for eco‐friendly pest control solutions.^[^ ^52^ ^]^ However, thujones, especially (α)‐Thujone, present notable toxicological risks, while the β‐diastereomer is generally considered less toxic. Acting as a gamma‐aminobutyric acid receptor antagonist, thujones can induce neurotoxicity at high concentrations, manifesting as seizures, hepatotoxicity, and nephrotoxicity.^[^ ^52^ ^]^ For regulatory purposes, the sum of both isomers is generally assessed, and the term thujone without specifying any isomer usually refers to the total content of both diastereomers. While historical claims of absinthe‐induced hallucinations due to thujone have been debunked, excessive consumption remains hazardous. Consequently, strict regulatory limits are imposed on thujone concentrations in consumable products to mitigate risks.^[^ ^52^ ^]^

Similarly, ***trans*** **‐sabinol**, the major component of Lt‐8, is a monoterpenoid alcohol and an intermediary in thujone synthesis and exhibits antimicrobial, antifungal, and antioxidant activities.^[^ ^52^ ^]^ However, toxicological studies have suggested that *trans*‐sabinol must be regulated with concentration limits to ensure safety. The FDA has included *Juniperus sabina* L. in the list of Poisonous Plant Database due to the presence of *trans*‐sabinol and its derivatives (sabinene, sabinol, and sabinyl acetate), which are associated with the responsibility for kidney congestion, hematuria, abdominal viscera congestion, menorrhagia, and abortion.^[^ ^53^ ^]^

In most cases, the main constituents of an EO that defines its chemotype are responsible for its bioactivity. These major compounds or their mixtures could be used to develop a typified *L. turbinata* EO, easily dosable for commercial purposes. However, isolated EO compounds at high concentrations have demonstrated cytotoxic and hepatotoxic effects, raising concerns about their safety when used excessively. Direct exposure to undiluted compounds may cause irritation to skin and mucous membranes, emphasizing the importance of adhering to safe usage levels and handling protocols, especially in consumer or therapeutic formulations use.^[^ ^54^, ^55^, ^56^ ^]^

Despite the focus on major constituents, minor compounds can also play a crucial role in the bioactivity of the EO.^[^ ^57^ ^]^ The synergistic interactions of the complex mixture of metabolites within an EO can enhance bioactivity and reduce the required doses of individual compounds. Thus, an EO is not solely defined by its major compounds but rather by a collective interaction of all its compounds.

After evaluating the distribution of populations across clusters using the four principal components, it was observed that most samples were grouped into cluster III, with only a few samples fully explained in clusters I and II. Given this imbalance, a broader analysis was conducted to explore a comprehensive distribution by incorporating additional components present in lower concentrations. To achieve this, the 11 compounds (limonene, 1,8‐cineole, piperitenone, piperitenone oxide, β‐caryophyllene, carvone, bornyl acetate, caryophyllene oxide, humulene epoxide II, borneol, and camphene) with major abundance and frequency of occurrence were selected to perform a new PCA analysis.

**Figure 4:**
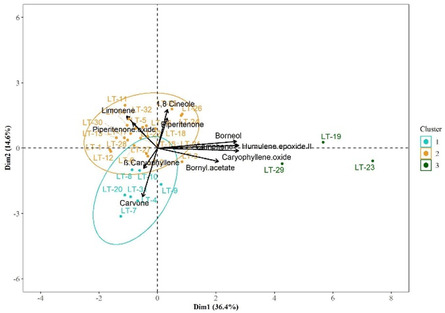
Cluster analysis using the hierarchical clustering on principal components (HCPC) algorithm for clustering *L. turbinata* populations including the 11 most frequent metabolites in the EO profiles.

The PCA analysis using the 11 compounds with the highest and medium relative frequencies of occurrence as variables accounted for 51.0% of the total variability in the system. This expanded analysis reclassified the populations into three new clusters (**Figure** **4**). Cluster I (Lt‐4, 7‐10, 20, and 31) was mainly defined by carvone and ß‐caryophyllene; Cluster II (Lt‐1‐3, 5‐6, 11‐18, 21‐22, 24‐28, 30, and 32) was dominated by limonene, piperitenone, piperitenone oxide, and 1,8‐cineole; and Cluster III (Lt‐19, 23, and 29) was characterized by contribution from the other major EO components.

In this context, compounds with intermediate relative frequencies, such as 1,8‐cineole, borneol, bornyl acetate, camphene, and humulene epoxide II, played important roles in defining the variability among the populations. **1,8‐cineole** (eucalyptol) is a monoterpene oxide with a camphoraceous, minty aroma widespread as a fragrance and flavor component. It is a prominent bioactive compound with diverse applications in the pharmaceutical, food, and cosmetic industries. It exhibits anti‐inflammatory, antimicrobial, antifungal, insecticidal antioxidant, neuroprotective, pro‐apoptotic, analgesic and expectorant, mucolytic/secretolytic, and bronchodilator properties, supporting its applications in respiratory and pain‐relief formulations^[^ ^58^ ^]^ and potential for the development of natural insecticides.^[^ ^59^, ^60^ ^]^ Given its extensive range of beneficial properties across multiple diseases, there has been a growing interest in exploring its mechanisms of action and potential therapeutic applications in various conditions, including respiratory, neurological, gastrointestinal, and oncological disorders. Recent studies have underscored the potential of 1,8‐cineole in addressing conditions such as Alzheimer's disease, neuropathic pain, and cancer.^[^ ^61^, ^62^ ^]^ Despite its wide‐ranging benefits, it must be used within defined safety limits to mitigate risks.

**Borneol**, a bicyclic monoterpene alcohol, and bornyl acetate, its ester derivative, are valued for their distinctive woody and camphoraceous aromas, as well as their diverse biological activities. Borneol exhibits antimicrobial, anti‐inflammatory, analgesic, and antioxidant activity. Both compounds have shown potential in improving cardiovascular health and supporting neurological functions, particularly cardio‐cerebrovascular diseases, with a crucial role in enhancing drug delivery and improving bioavailability. Additionally, borneol is widely utilized in the food, daily chemicals, fragrances, and flavors industries.^[^ ^63^, ^64^ ^]^ Similarly, bornyl acetate, known for its pleasant, balsamic, and camphoraceous aroma, is widely used in the fragrance, flavor, and pharmaceutical industries and exhibits promising pharmacological properties, especially anti‐inflammatory and immunomodulatory effects.^[^ ^65^ ^]^

**Camphene**, a bicyclic monoterpene with a pungent odor, exhibits significant antioxidant and anti‐inflammatory activities, contributing to its potential in formulations aimed at protecting skin health, managing respiratory conditions, and serving as natural preservative in food and cosmetic products. Numerous in vitro and in vivo studies have demonstrated the biological properties of camphene, including antibacterial, antifungal, anticancer, antioxidant, antiparasitic, antidiabetic, anti‐inflammatory, and hypolipidemic activities. Moreover, camphene has been reported to exhibit antileishmanial, hepatoprotective, antiviral, and antiacetylcholinesterase inhibitory activities.^[^ ^66^ ^]^ Camphene also demonstrated eco‐friendly insect‐repellent capabilities, supporting its use in sustainable pest management strategies.^[^ ^67^ ^]^ Camphene is considered safe for use in food, cosmetic, and therapeutic applications within established concentration limits.

Finally, **humulene epoxide II**, a sesquiterpene derivative of humulene (an isomer of caryophyllene) with earthy, herbal aroma, exhibits antimicrobial, antifungal, and anti‐inflammatory properties.^[^ ^68^ ^]^

As stated by Catalán et al. (2021),^[^ ^15^ ^]^ the chemical composition of EO of the different collections shows remarkable qualitative variations.

In six collections (Río Cuarto‐Córdoba,^[^ ^26^ ^]^ Altas Cumbres^−^ Córdoba,^[^ ^27^ ^]^ Los Llanos—La Rioja,^[^ ^21^ ^]^ San Luis,^[^ ^19^ ^]^ Merlo‐San Luis,^[^ ^20^ ^]^ and Salta^[^ ^25^ ^]^), the dominant component was limonene; α‐thujone was the main component in three collections (Saldán‐Córdoba,^[^ ^18^ ^]^ Colón‐Córdoba,^[^ ^17^ ^]^ and Quebrada de Olta‐La Rioja^[^ ^23^ ^]^); carvone in two (side of the road, national highway 38‐La Rioja,^[^ ^22^ ^]^ and Roque Sáenz Peña‐Chaco^[^ ^28^ ^]^); piperitenone oxide was the main component in one (Valle de Atinaco, Los Colorados—La Rioja^[^ ^24^ ^]^) and the second component in two collections (San Luis^[^ ^19^ ^]^ and Merlo‐San Luis^[^ ^20^ ^]^); and *trans*‐sabinol (Córdoba^[^ ^29^ ^]^) and caryophyllene oxide (Los Molinos‐Córdoba^[^ ^29^ ^]^) in one collection each (Figure 1).

**Figure 5:**
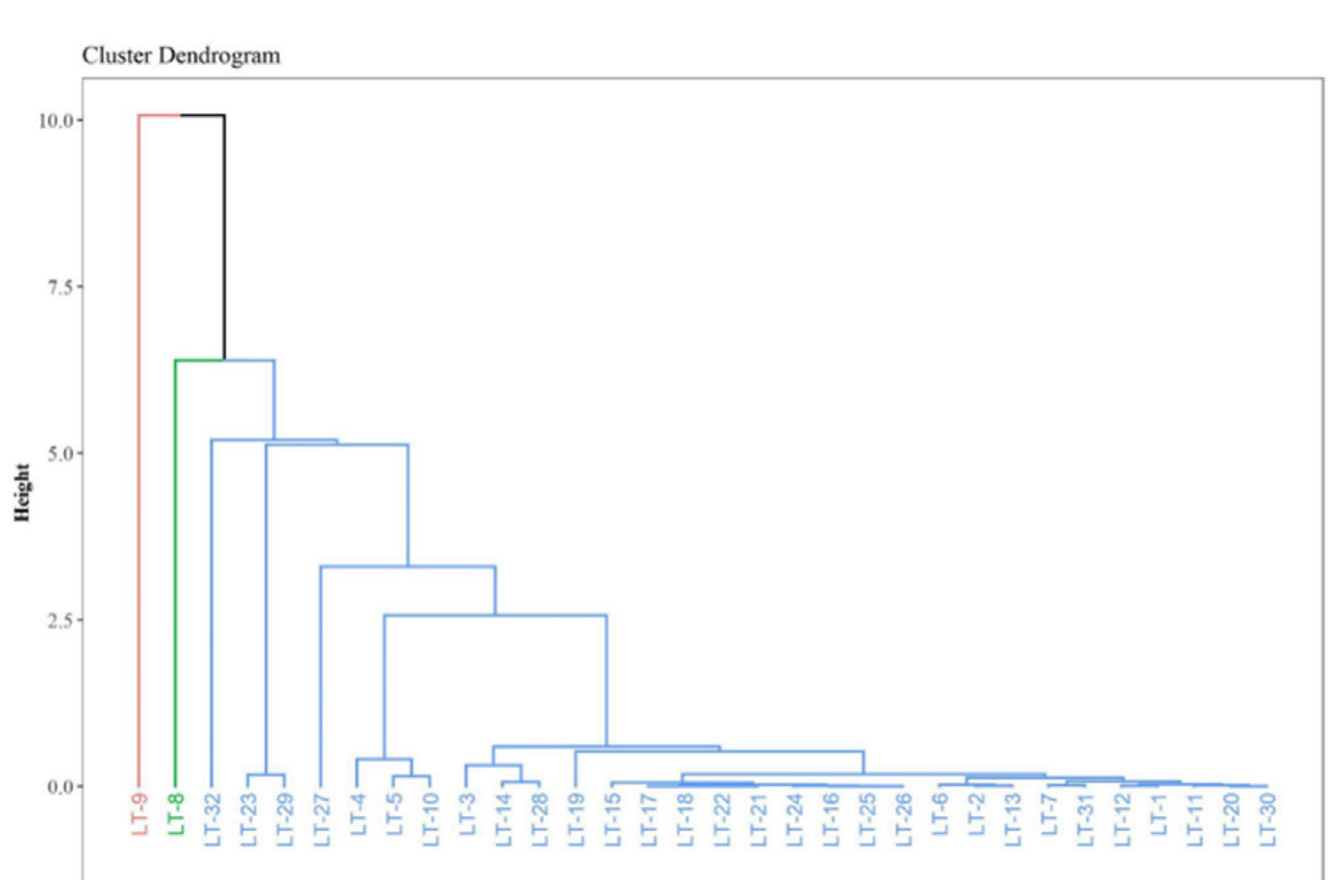
Cluster analysis based on Euclidean distances of EO components from different populations of *L. turbinata*, incorporating minor and major metabolites present in their EOs.

The EO of *L. turbinata* highlights the existence of chemotypes among several populations of a given species. Linear discriminant and cluster analyses (dendrograms) based on Euclidean distances of EO compounds have proven to be valuable statistical tools.^[^ ^8^ ^]^ However, when cluster analysis is performed using all metabolites present in the EO profiles of *L. turbinata* (**Figure** **5**), the groupings become less intuitive due to the complexity introduced by minor components being present in some populations but absent in others.

*L. turbinata* exhibits high variability in EO composition, a characteristic shared with other species of the Verbenaceae family, such as *Aloysia citriodora* Palau,^[^ ^69^ ^]^ *Aloysia gratissima* (Gillies & Hook.) Tronc.,^[^ ^70^, ^71^, ^72^, ^73^ ^]^ *Salimenaea integrifolia* (Griseb.) N. O'Leary & P. Moroni,^[^ ^74^ ^]^ *L. alba* (Mill.) N. E. Br. ex Britton & P.Wilson,^[^ ^75^ ^]^ *L. junelliana* (Moldenke) Tronc.,^[^ ^76^ ^]^ *L. multiflora* Moldenke,^[^ ^77^, ^78^ ^]^ and numerous other *Lippia* species.^[^ ^15^, ^16^, ^79^ ^]^ This diversity underscores the existence of a wide range of chemotypes within the family.

Almeida et al. (2018)^[^ ^79^ ^]^ conducted a PCA analysis of EO composition in *L. origanoides* Kunth, *L. origanoides* × *velutina*, *L. velutina*, *L. sidoides*, and *L. salviifolia* from diverse geographical locations. Their analysis revealed no species‐specific clustering, suggesting that these taxa may belong to a single species and exhibit high chemovariability. This hypothesis was later supported by amplified fragment length polymorphism and Internal Transcribed Spacer molecular data, leading to the proposal that these species should be treated as synonyms. Similarly, Tozin et al. (2015)^[^ ^80^ ^]^ reported considerable intraspecific variability in *L. origanoides*, both among individuals from different populations and within the same population.

In *L. alba,* such variability has been attributed to a complex interplay of factors, including genotype,^[^ ^75^ ^]^ ploidy level, and environmental conditions such as water stress.^[^ ^6^ ^]^ Furthermore, Ricciardi *et al.* (2011)^[^ ^69^ ^]^ demonstrated that differences in the EO chemical composition of wild and cultivated *A. citriodora*, despite sharing the same genetic origin, could not be explained by environmental or seasonal factors alone. Collectively, these findings emphasize the strong genetic basis underlying EO variability in Verbenaceae species.

To date, only Coronel et al. (2005)^[^ ^22^ ^]^ have analyzed tetraploids (2 n = 60) collections of *L. turbinata* Griseb. f. *magnifolia* Moldenke (= *Lippia fissicalyx* Tronc.). Their findings revealed that the EOs of these collections were typically dominated by two main components, which together accounted for more than 75% of the oil: limonene and either piperitenone oxide (= lippione), carvone, or 1,8‐cineole. In contrast, both diploid (2 n = 30) and tetraploid (2 n = 60) collections of *L. turbinata* Griseb. f*. turbinata* showed that tetraploid individuals from Catamarca province shared chemical similarities with some *L. turbinata* Griseb. f. *magnifolia* Moldenke collections. These results suggest that the EO from tetraploids are more chemically diverse and complex, likely due to their greater cytogenetic variability and enzyme polymorphism.^[^ ^22^ ^]^

According to Catalan *et al.* (2021),^[^ ^15^ ^]^ the chemical composition of the EO obtained from the same plant stock of *L. turbinata* remains essentially stable over several years, indicating that the EO profile is predominantly under genetic control.

## 4 Essential Oil Variations and Correlation with Biosynthetic Pathways

As previously explained, in the species *L. turbinata*, several main components have been identified, including limonene, carvone, thujone, piperitenone, caryophyllene, and their oxide derivatives. This combination of principal components may be crucial for describing possible chemotypes, which are strongly linked to genetic factors within the species. However, it is important to note that variations in the proportion of these components could also be influenced by factors such as harvesting time, EO isolation processes, and chemical instability caused by oxidative agents during sample handling and storage. Among the samples studied, limonene was the predominant component, often combined with other principal components such as piperitenone/piperitenone oxide, carvone, thujone, and caryophyllene/caryophyllene oxide. The most abundant combination, limonene/piperitenone, is represented by numerous populations in Argentina. This biosynthetic pathway has been described in mint (*Mentha spp.*), where limonene undergoes several biosynthetic steps to form pulegone and menthone, with (−)‐isopiperitenone acting as an intermediate compound.^[^ ^48^ ^]^ This intermediate serves as a precursor for monoterpene epoxyketones, including *trans*‐piperitone oxide and piperitenone oxide. In mint, the biosynthesis of these compounds involves the isomerization of isopiperitenone to piperitenone by isopulegone isomerase, followed by cytochrome P450‐dependent epoxidation of piperitenone to (+)‐piperitenone oxide. This is subsequently reduced by pulegone reductase, which is NADPH‐dependent, to form (−)‐*trans*‐piperitone oxide. In the case of *L. turbinata*, the enzyme (+)‐*cis*‐isopulegone isomerase may be highly expressed, thereby increasing the biosynthesis of piperitenone and its derivatives, as observed in major populations. Another biosynthetic pathway observed in this species leads to the production of α‐ or β‐thujone as the final metabolite after four steps. In this pathway, geranyl diphosphate serves as the precursor, producing sabinene as the first terpene. This step is catalyzed by the enzyme sabinene synthase (SS). Sabinene is subsequently converted to its alcohol, sabinol, in both *cis*‐ and *trans*‐configurations. Sabinone is then formed through an NADPH‐ and oxygen‐dependent dehydrogenase. Finally, an NADPH‐dependent stereoselective reduction produces the α‐ or β‐thujone isomers.^[^ ^52^ ^]^ Similarly, the synthesis of 1,8‐cineole, borneol, camphor, and their derivatives is regulated by enzymes analogous to SS, such as 1,8‐cineole synthase and (+)‐bornyl diphosphate synthase.^[^ ^81^ ^]^ These pathways have been described and studied in species such as *Tanacetum vulgare* L., *Salvia officinalis* L., *Artemisia absinthium* L., and *Thuja plicata* Donn ex D. Don. The regulation of these pathways can be influenced by a single genomic locus, as in *T. plicata*, or by transcriptional control, as in *S. officinalis*, where no direct correlation has been found between mRNA levels and the concentration of sabinene end products, reflecting more complex genetic and metabolic regulation.^[^ ^81^ ^]^

Another terpene of interest, commonly found in many aromatic plants, including the *Lippia* genus, is the sesquiterpene caryophyllene and its derivatives, along with the related compound α‐humulene. In plants, the biosynthetic pathway for β‐caryophyllene and α‐humulene is thought to originate from the cyclization of a farnesyl pyrophosphate precursor into an (E,E)‐humulyl carbocation, catalyzed by sesquiterpene cyclase. Following cyclization, (E)‐β‐caryophyllene is further converted into a caryophyllenyl cation, characterized by a bicyclo structure with 4‐ and 9‐membered rings. From this precursor, isocaryophyllene is produced through enzymatic E‐Z isomerization, while β‐caryophyllene oxide derives from 4‐5 oxidation of β‐caryophyllene.^[^ ^39^ ^]^

After describing the various possible biosynthetic pathways in the defined populations, it is important to highlight that the concentration of certain metabolites—such as piperitenone, sabinol, thujene, bornyl acetate, and caryophyllene or their derivatives—could be directly linked to enzyme activities influenced by factors such as weather conditions, season, ontogenic regulation, or external variables such as isolation procedures, methodologies, and distillation time. However, determining a specific chemotype and its corresponding population is complex and challenging.

For example, a study on the effects of harvesting time and hydrodistillation on mint EO demonstrated that the chemical profile can vary depending on the time of harvesting. The highest menthol enrichment was observed at 9:00 a.m., whereas the highest menthone concentration was recorded at 5:00 p.m. This inverse relationship between menthol and menthone concentrations may also be attributed to menthol oxidation during hydrodistillation.^[^ ^82^ ^]^

Therefore, a thorough evaluation of the possible chemotypes of *L. turbinata* is necessary, considering variables such as harvesting time, collection material, season, ontogenic stage, hydrodistillation methods, and procedure time, as well as other internal parameters. Further research into the chemical profile of *L. turbinata* is required to better understand its biosynthetic pathways and chemotypic variations.

## 5 Uses and Applications of *Lippia turbinata* Essential Oils

Species of the *Lippia* genus, including *L. turbinata* (commonly known as poleo), produce a wide array of volatile bioactive compounds that contribute to their diverse pharmacological activities and potential synergistic effects. The chemical composition, pharmacological activities, and ethnobotanical uses of various *Lippia* species have been extensively documented in the literature.^[^ ^12^, ^15^, ^16^ ^]^

The EO of *L. turbinata* is characterized by a high concentration of limonene and has demonstrated remarkable antifungal activity. It has been shown to inhibit phytopathogenic fungi such as *Sclerotium rolfsii*, *S. sclerotium*, and *Rhizoctonia solani*,^[^ ^25^ ^]^ as well as against *Aspergillus spp.*,^[^ ^26^ ^]^ and the ascomycete fungus *Ascosphaera apis*.^[^ ^19^ ^]^ Furthermore, the EO exhibits potent nematicidal properties against root‐knot nematodes (*Meloidogyne sp.*).^[^ ^20^ ^]^

In addition to its antifungal and nematicidal activity, *L. turbinata* EO possesses notable antioxidant properties^[^ ^24^, ^27^, ^83^ ^]^ and broad‐spectrum antimicrobial effects. It has demonstrated efficacy against various microorganisms, including yeasts, bacteria,^[^ ^28^, ^83^, ^84^ ^]^ *Paenibacillus larvae*,^[^ ^29^, ^85^, ^86^ ^]^ and fungal pathogens associated with peanut seed diseases.^[^ ^87^, ^88^ ^]^

Notably, the EO also exhibited insecticidal activity against vectors and pests such as *Culex quinquefasciatus* ^[^ ^23^, ^89^ ^]^ and *Musca domestica* ^[^ ^90^ ^]^ and showed repellent effects against the parasitic mite *Varroa destructor*.^[^ ^91^ ^]^ These properties make *L. turbinata* EO a promising candidate for use as a botanical pesticide.

The application of EOs and their individual constituents in biopesticides presents several advantages. These natural compounds generally degrade into nontoxic components, significantly reducing their environmental impact and toxicity to nontarget organisms. However, despite these benefits, only a limited number of EO‐based biopesticides have reached the market. Several challenges remain, particularly regarding the stability and efficacy of EOs under varying environmental conditions.^[^ ^92^ ^]^ Moreover, isolated EO constituents must undergo rigorous evaluation to determine their safety profiles, especially concerning environmental residues and appropriate dosage levels. The widespread reliance on synthetic pesticides has led to increased resistance among pest species, ecological disruption, bioaccumulation in food chains, and health risks to agricultural workers and consumers.^[^ ^93^ ^]^ In this context, *L. turbinata* EO offers a compelling alternative due to its proven bioactivity and relatively low toxicity. Several studies have confirmed its effectiveness in pest control applications while suggesting that it is less harmful than conventional synthetic chemicals.^[^ ^23^, ^31^, ^89^, ^94^ ^]^

Importantly, *L. turbinata* EO is less persistent in the ecosystem, which contributes to its sustainability and ecological compatibility. As a part of a broader movement toward greener pest management strategies, research continues to explore the integration of EO components in agricultural practices. These efforts focused on exploiting the natural biodegradability and low toxicity of EO compounds to develop safer, eco‐friendly pest control methods.^[^ ^92^ ^]^

Beyond its pesticidal potential, *L. turbinata* EO exhibited additional bioactivities. It has shown virucidal effects against the Junin virus and some degree of activity against herpes simplex virus type 1 (HSV‐1) and dengue virus type 2 (DEN‐2).^[^ ^95^ ^]^ Furthermore, García et al. (2018)^[^ ^32^, ^33^ ^]^ reported properties which could be relevant for mitigating methane emissions in livestock farming.

However, caution is advised regarding the internal use of *L. turbinata* EO. According to Fester et al. (1960),^[^ ^96^ ^]^ one of its major constituents, piperitenone oxide (lippione), and its derivative diosphenolene (lippiaphenol) have been shown to stimulate uterine motility. Therefore, consumption of the oil is contraindicated during pregnancy.^[^ ^97^ ^]^

## 6 Summary and Outlook

Populations of *L. turbinata* from different geographic regions exhibited substantial variability in their EO profiles. This variability can be attributed to intrinsic characteristics, such as genetic factors (e.g., ploidy level), as well as interactions with environmental factors. Additionally, qualitative and quantitative chemical traits are influenced by variables such as extraction methodologies, the phenological stage of the plants at collection, drying and storage methods, harvesting/climatic conditions, and other factors that can alter EO composition.

To definitively classify the chemotypes of *L. turbinata*, comparative studies using unsupervised classification algorithms are recommended. These studies should standardize key factors and incorporate additional analyses, such as chromatographic profiling of nonvolatile secondary metabolites. Such approaches would enable robust multivariate statistical analyses to compare EO profiles across populations, identify critical thresholds for chemotype differentiation, and facilitate the selection of specimens optimized for specific applications in food, pharmacology, or agriculture.

The EOs of *L. turbinata* are particularly valuable due to their richness in bioactive compounds with diverse applications, such as medicinal, food, and agricultural (e.g., eco‐friendly pest control). The ability of *L. turbinata* to provide EOs with structurally diverse and biodegradable compounds makes them a promising agroecological alternative to synthetic chemicals. Furthermore, these EOs could serve as effective replacements or complements to synthetic compounds in the chemical industry, avoiding many of the undesirable side effects associated with the synthetic counterparts.

To maximize the safe and effective use of *L. turbinata* EO‐based preparations, further systematic research is needed to investigate the factors driving EO variability across different geographic regions. Such studies will contribute to the sustainable exploitation of this species for its chemical and biological potential in diverse industrial applications.

This study highlights the significant chemical variability in the EO profiles of different populations of *L. turbinata*. Some populations exhibited more desirable profiles of major constituents depending on their intended applications. The EOs are best characterized by the four major constituents, whose relative frequencies and abundances provide a basis for typifying both the EOs and their populations. However, further standardized analyses and cultivation under controlled conditions are necessary to better understand the causes of this variability and the behavior of the EOs under various environmental and agronomic conditions.

## Conflict of Interest

The authors declare no conflict of interest.

## Supporting information

Supplementary Material
